# Assessment of Soil Carbon Stock and Soil Quality in Different Forest Stands and Management Regimes in Terai Region of Nepal

**DOI:** 10.1155/2024/1739115

**Published:** 2024-11-15

**Authors:** Durga Kandel, Sachin Timilsina, Santosh Ayer, Saroj Kumar Chaudhary, Jeetendra Gautam, Rabindra Adhikari, Kishor Prasad Bhatta

**Affiliations:** ^1^Department of Soil and Watershed Management, Institute of Forestry, Tribhuvan University, Pokhara Campus, Pokhara 33700, Nepal; ^2^Department of Forest Biometrics, College of Natural Resource Management (CNRM), Agriculture and Forestry University, Katari 56310, Nepal; ^3^Department of Soil, Forest Research and Training Centre (FRTC), Babarmahal, Kathmandu 44600, Nepal; ^4^Department of Forest Survey and Engineering, Faculty of Forestry, Agriculture and Forestry University, Hetauda 44107, Nepal; ^5^Department of Forest Research, Research and Development Centre, Kathmandu 44600, Nepal

**Keywords:** forest regimes, forest soil, soil properties, soil quality index, terai forest

## Abstract

Assessment of soil organic carbon (SOC) stock and soil quality for informed forest management is hindered by inadequate data across different forest stand types and management regimes. Therefore, this study was conducted to assess SOC stock and soil quality in two forest stand types, i.e., *Shorea robusta* (Sal) forest and Terai mixed hardwood (TMH) forest, and selected forest management regimes (leasehold forest, community forest, government-managed forest, and forest area under protected area) in Terai region of Nepal. Stratified random sampling method was adopted for soil sample collection across terai region following Forest Resource Assessment, Nepal. Altogether, 62 composite soil samples from 30 cm depth were taken from the entire Terai region which included these two forest stand types and four management regimes. Different physical (soil texture and bulk density) and chemical (pH, SOC (%), total nitrogen, available phosphorus, and available potassium) properties were analyzed to calculate SOC stock and soil quality. Our result found no significant differences in SOC stock among two forest stand types (*p* > 0.05). Unexpectedly, leasehold forest had significantly (*p* < 0.05) higher SOC stock than other forest management regimes. In terms of soil quality, among two forest stand types, Sal forest (0.50) was found to be superior compared with TMH forest (0.46). Similarly, community forest had superior soil quality (0.50) than government-managed forest (0.47), protected area (0.47), and leasehold forest (0.45). A longitudinal study approach is recommended to observe changes in soil properties over time due to climate change and human activities, offering valuable insights into their dynamics.

## 1. Introduction

The term “soil quality” refers to inherent ability to efficiently supply various ecosystem services including the maintenance or enhancement of water and air quality, as well as the provision of a safeguarded atmosphere for human health and livelihood [1, 2]. Soil quality is a complex interaction of numerous physiochemical and biological traits that undergo change by external variables such as utilization and management of land, surrounding environmental conditions, and socioeconomic concerns [3]. It is influenced by various soil physical properties such as soil texture, structure, and porosity, which impacts functions such as water infiltration, root growth, and soil erosion [4]. Chemical properties, including soil pH, nutrient levels, and organic matter content, have also major impact on soil quality, affecting nutrient availability, soil fertility, and ultimately affects health condition of soil [5, 6]. Similarly, biological properties are also essential for the disintegration of organic materials, cycling of nutrients, and preservation of soil structure [7]. It is important to acknowledge the interrelationships and interdependencies among these soil properties, as alterations in one factor can cascade and impact other aspects, ultimately influencing soil quality [8]. Therefore, a comprehensive understanding and evaluation of these factors are essential for effective soil management, sustainable land utilization practices, and the preservation of soil health and productivity.

Globally, numerous studies have underscored the critical links between soil quality, forest health, and the ecosystem services provided, such as water purification, carbon storage, and habitat preservation [9–15]. Healthy soils are fundamental to the functioning of ecosystems, influencing plant growth, regulating nutrient cycles, and supporting diverse organisms. Soil organic carbon (SOC), a key indicator of soil quality, plays a pivotal role in carbon sequestration, mitigating the effects of climate change by storing large amounts of carbon that would otherwise contribute to atmospheric CO_2_ levels [16]. For instance, the Food and Agriculture Organization (FAO) [17] highlighted the significant role that healthy soils play in global carbon sequestration and climate regulation. The degradation of soil quality can disrupt these processes, leading to diminished ecosystem services, loss of biodiversity, and reduced forest productivity. Additionally, the loss of soil health often results in increased soil erosion, which can impact water quality and lead to land degradation on a broader scale [18]. These global perspectives underscore the importance of understanding and maintaining soil quality for the long-term sustainability of ecosystems. As forests cover about 31% of the world's land area, their role in maintaining healthy soils is critical not only for local environments but also for the global carbon balance as well [19]. Healthy soils contribute to the stability of forest ecosystems, enhancing their resilience against disturbances such as deforestation, climate change, and human interventions. Furthermore, global initiatives, such as the United Nations Sustainable Development Goals (SDGs), emphasize the need for sustainable soil management practices to support biodiversity, food security, and climate resilience on a global scale [19].

South Asian countries, particularly Nepal, with its unique geographical features and forest ecosystems, present a compelling case for studying soil quality. Nepal is characterized by different forest types, including pure Sal (*Shorea robusta*) forests^1^, Sal mixed forests^2^, bamboo forests, pine forests,^3^ and others [20–23]. In Terai region of Nepal, pure Sal forests are dominated by Sal trees and form dense, closed-canopy forests whereas Terai mixed hardwood (TMH) consists of a blend of tree species, often including Sal trees, but with a higher diversity of other broadleaf species [21]. These forests provide critical ecosystem services, support local livelihoods, and contribute to biodiversity conservation [24]. Since these forests are characterized by variations in species composition, vegetation structure, and environmental conditions [25], soil quality can vary significantly [26]. Similarly, the forests of Nepal are broadly classified as “private forest” and “national forest” on the basis of ownership. National forests are further disaggregated into five categories; protection forest, community forest, leasehold forest, religious forest, and government-managed forest [27]. Community forestry (CF) has been accorded the highest-priority forestry sector and has widely been acclaimed as a successful forest management regime in Nepal [28]. A pilot survey showed that community-managed forests sequester substantial amounts of carbon [29]. Many successful studies have claimed that community-managed forests offer Nepal's substantial carbon sequestration potential [30]. At the same time, the protected forest is a relatively new approach to forest management in Nepal [28]. Such management systems under different forest and land management types lead to undesirable change in the soil structure [31], decline in the SOC stocks, soil fertility, soil productivity [32, 33], and ultimately soil quality [34].

Maintaining and enhancing soil quality is a high concern in developing nations like Nepal, where a large portion of the population continues to rely on woodlands and farming [35]. As the improper management of soil can result in detrimental changes in soil function, appropriate tools and methods are required for assessing and monitoring soil quality [36]. Numerous studies in Nepal have been conducted [3, 35, 37]; however, they primarily concentrated on evaluation of soil quality of farmland and agricultural land as well as just on single forest types. Also, a great deal of information is available on how land use and management influence SOC, which is considered proxy for soil quality [38]. Previous studies in Nepal have focused on assessing effect of agroforestry types [39], silvicultural treatments [40], and different land use types on SOC stock and soil quality [35]. However, information is scanty on the influence of forest stand types and management regimes on SOC stock and soil quality. Therefore, to address this research gap, this study was conducted to assess the impact of different forest stand types and management regime on SOC stock as well as soil quality in Terai region of Nepal. We hypothesize that the TMH forest stand type and community-managed forest management regime have significantly higher SOC stocks and soil quality than other forest types and management categories.

## 2. Materials and Methods

### 2.1. Site Description

**Figure 1:**
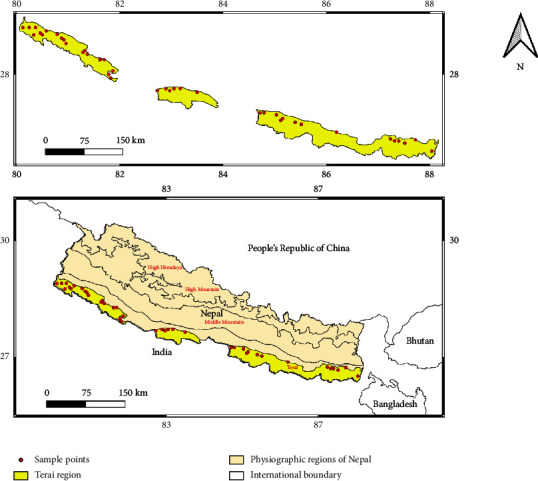
Map showing study site, i.e., Terai region of Nepal with sample plots.

This study was conducted in the Terai region (80°4′30″ to 88°10′19″ E longitude and from 26°21′53″ to 29°7′43″ N latitude) of Nepal (Figure 1). The Terai covers 2,016,998 ha, gently sloping at rates of 2–10 m/km [21]. The population in this area is 26,494,504 [41], and the elevation ranges from 63 to 330 m above mean sea level (msl) [43]. The Terai region predominantly features alluvial soil with varying textures from sandy to clay. Approximately 76.45% of the Terai's total forests are located outside protected areas, 16.97% within protected areas, and 6.57% in the buffer zone. Among the total forest area of 411,580 ha in the Terai region, *Shorea robusta* (Sal) forests constitute 188,133 ha, while TMH forests, including species such as *Terminalia alata*, *Mallotus philippensis*, *Adina cordifolia*, *Lagerstroemia parviflora*, and *Anogeissus latifolius*, cover 192,866 ha [43]. The Terai region has a subtropical climate with hot, muggy summers, heavy monsoon rains, and dry winters. April and May experiences the highest monthly mean temperatures of 35°C–40°C, while January sees the lowest monthly mean temperatures of 14°C–16°C. From east to west, annual precipitation drops from 2680 mm to 1138 mm [42]. The majority of pure Sal forest plots are found in community forests, with some government-managed forests, and a few in protected areas. Similarly, most TMH plots are located in government-managed forests and protected areas, with some in buffer zones and community forests (Figure 1). Studied forest stand types under different management regimes are naturally regenerated forest. Different management regimes such as community forest, leasehold forest, protected areas, and government-managed forests are managed differently in Nepal. In Nepal, while harvesting and timber extraction are generally prohibited in protected areas, activities such as thinning dense forests, removing invasive species, and controlled burning are implemented for wildlife habitat management. Leasehold forestry empowers poor households to utilize degraded lands for agroforestry, improving their livelihoods. Community forests are managed using various silvicultural systems, like irregular shelterwood and selection systems, to provide timber and ecosystem services, while government-managed forests address timber demands not met by other regimes.

### 2.2. Sampling Design

This study adopted stratified random sampling method for soil sample collection following Forest Resource Assessment in Nepal [44, 45]. There were 162 permanent plots laid by Department of Forest Research and Survey (DFRS) for the periodical forest resource assessment of these forest types (80 for Sal forest and 82 for TMH forest). We selected 62 plots (31 in each category randomly) for this study ensuring the sample from all parts of Nepal, i.e., eastern, western, and central Nepal. Plots in all available management regimes were also considered which resulted in 6, 15, 19, and 22 plots in leasehold forest, protected area, government-managed forest, and community forest, respectively. Further, the distance of the permanent plots from the available trail/highway was also considered as the selection criteria. Detail information regarding sample plots is shown in Table 1

### 2.3. Soil Sampling

**Figure 2:**
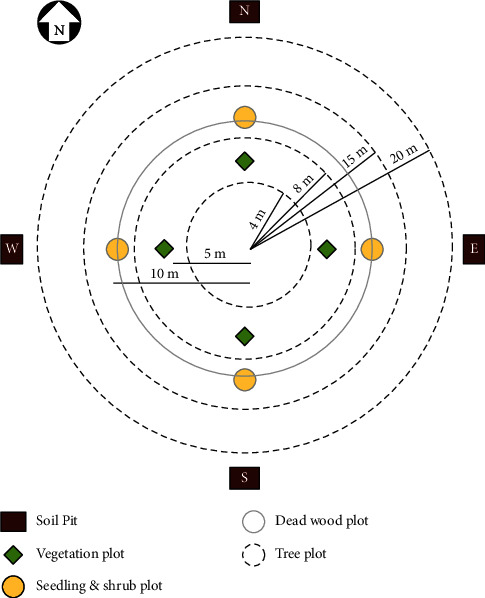
Plot layout for soil sample collection; source [21, 46].

Soil samples were obtained from four subplots within each plot, including samples from each specified pit unless the designated pit was identified as farmland, an extremely steep incline (>100%), a riverbank, a roadway, a rocky terrain, or in the vicinity of a water body. At least three soil sampling points were selected within each subplot (Figure 2). Then, 62 composite soil samples (31 from each stand type) upto 30 cm depth were gathered from all soil pits, excavated within a 2∗2m area, positioned 1 m beyond the 20 m plot radius (Figure 2). We focused on analyzing the soil quality of the top 0–30 cm layer because beyond 30 cm and down to 1 m, the soil parameters tend to exhibit less variability as we delve deeper into the forest soil layers [47].

### 2.4. Soil Sample Analysis

SOC was determined by Walkley and Black method [48]. It is most commonly used in tropical countries, because of its cost-effectiveness and simplicity of the procedure [49]. However, it may underestimate results if not properly calibrated or in the presence of interfering substances [50]. Olsen's and Somers method [51] was used to determine available phosphorus (P) whereas flame photometer method [52] was adopted to determine available potassium (K). These methods are widely used and validated standard technique because it specifically targets phosphorus and potassium that is readily soluble and available for plant uptake. However, it may not account for forms that are tightly bound to soil minerals or organic matter potentially underestimating total availability [53]. Total nitrogen (N) was determined by Kjeldahl method [54]. Although this method is widely used, it is time-consuming and susceptible to interference from other compounds such as nitrates and nitrites which can affect accuracy [55]. Soil texture was determined by Bouyoucos hydrometer method [56]. It is rapid and simple method which is suitable for finer soil particles but may encounter challenges with coarser textures, potentially leading to inaccuracies [57]. Bulk density (BD) was measured using oven dry method [58] which is widely employed due to its simplicity and accessibility. However, its results can be affected by soil structure and moisture content variations, leading to potential variability in measurements [59]. Soil pH was measured with a digital pH meter [60]. It provides quick and reliable measurement of soil pH, but accuracy can be influenced by soil moisture levels, temperature, and electrode maintenance [61].

### 2.5. Calculation of Soil Carbon Stock

The SOC stock was determined based on soil depth, bulk density, and the percentage of SOC using the following formula:

(1) $$\begin{array}{c} \text{SOC stock}=\text{BD}\times d\times \text{SOC }\left(\%\right), \end{array}$$

where SOC stock represents the SOC stock per unit area (ton·ha^−1^), BD is the soil bulk density (g·cm^−3^), *d* is the depth of the sampled soil layer in centimeters (cm), and SOC (%) is the percentage of organic carbon.

### 2.6. Computation of Soil Quality

As direct measurement of soil quality is not feasible, it is deduced from various soil properties and quantified as the soil quality index (SQI) [62–64]. The assessment method of SQI is a widely used analytical method in the quantitative evaluation of soil quality in recent years [13]. Understanding SQI in forests is crucial as it offers a comprehensive assessment of soil health by integrating various parameters [65]. This numerical index simplifies interpretation and facilitates monitoring of soil degradation caused by land-use changes and management practices [66]. There are various approaches to estimate SQI such as using an additive system based on common soil parameters [67, 68], soil fertility/nutrient/index approach [69, 70], and statistical model-based SQI [71, 72]. In this study, we adopted additive system based on common soil parameters method for SQI computation due to its consistency of the outcomes generated which is also suggested by Abdu et al. [71] for soil quality assessment. The procedure consisted of three key stages: (i) selecting relevant indicators; (ii) translating indicators into scores; and (iii) integrating the scores to form an index [70, 73]. Scoring method was applied to interpret the SQI, as outlined in Table 2 [75]. Bajracharya et al. [74] assigned weight values to NPK in their equation, relying on the soil quality rating provided by NARC [76] (Table 3). The following formula was used to calculate SQI value [71, 74]:

(2) $$\begin{array}{c} \text{SQI}=\left[\left(a\times R_{\text{STC}}\right)+\left(b\times R_{\text{pH}}\right)+\left(c\times R_{\text{OC}}\right)+\left(d\times R_{\text{NPK}}\right)\right], \end{array}$$

where C, clay; Si, silt; S, sand; LS, loamy sand; CL, clay loam; SiL, silty loam; SC, sandy clay; SiCL, silty clay loam; SiL, silty loam; SiC, silty clay; SL, sandy loam; SCL, sandy clay loam; LS, loamy sand; SQR, soil quality rating.

### 2.7. Data for Environmental Variables

The precipitation and temperature data of the study area were derived from ERA5 monthly aggregate data provided by ECMWF/Copernicus Climate Change Service [77]. It provides the monthly average of air temperature in Kelvin scale and total precipitation (monthly sums) in meter from 1979 to 2020, which was later converted to monthly average temperature in degree Celsius and annual average precipitation in mm. Moreover, the elevation, slope, and aspect of the study sites will be obtained by using the shuttle radar topography mission (SRTM) digital elevation model (DEM) with a resolution of 90 m (https://srtm.csi.cgiar.org/srtmdata/). All environmental variables were processed in Google Earth Engine [78].

### 2.8. Statistical Analysis

All data statistical analyses were performed using Microsoft Excel 2010 and R studio version 4.2.3. Before statistical analysis, data were tested for normality (*p* > 0.05), and data were found to be normally distributed. Welch t-test was used to assess the statistically significant differences among soil carbon stock and other soil parameters under each forest type. Similarly, one-way ANOVA was used to assess the statistically significant differences among soil carbon stock and other soil parameters under each forest management regimes. Moran's I test was used for checking against spatial autocorrelation of the soil properties in the selected plots. Bulk density, total N, and available K exhibited significant spatial clustering, while available P and pH demonstrated spatial randomness (Table 4). Principle component analysis (PCA) was used to determine the relationships between environmental factors and soil properties in R Studio with “factoextra” package [79]. All our input variables were transformed into a 0–1 scale before running PCA [80].

## 3. Results

### 3.1. By Forest Stand Types

#### 3.1.1. Soil Characteristics of Forest Stand Types

**Figure 3:**
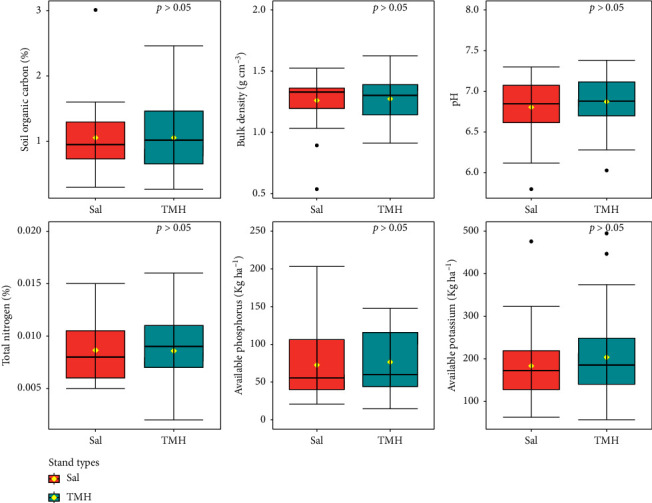
Soil properties in pure Sal (*Shorea robusta*) forest and Terai mixed hardwood (TMH) forest.

Soil texture in both Sal forest and TMH forest was found to be sandy loam and silty clay loam type. Mean SOC was found slightly higher in TMH forest (1.09%) than Sal forest (1.02%). Soil pH in Sal forest (6.83) and TMH forest (6.85) was found to be acidic in nature. Similarly, Sal forest soils were high in available K (200.99 kg·ha^−1^) and available P (87.38 kg·ha^−1^) than in TMH forest (188.46 kg·ha^−1^ available K and 68.01 kg·ha^−1^ available P) whereas total N content (0.01%) was found to be similar in both forests. BD was found to be almost similar in Sal (1.268 g·cm^−3^) and TMH (1.266 g·cm^−3^) forest. However, there were no statistically significant differences in soil properties among forest stand types (*p* > 0.05) (Figure 3).

#### 3.1.2. SOC Stock of Forest Stand Types

**Figure 4:**
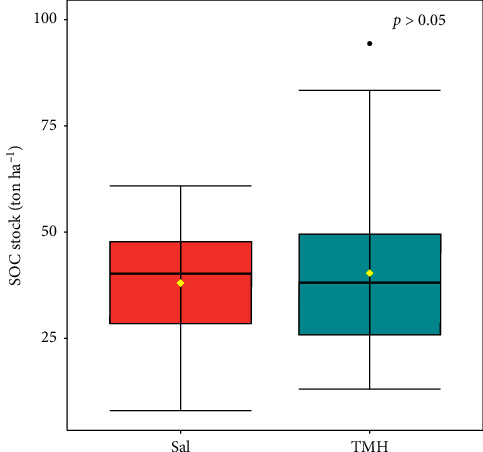
SOC stock of two forest stand type.

Higher SOC stock was found in TMH forest (40.40 ton·ha^−1^) than in Sal forest (38.06 ton·ha^−1^). However, this difference was not statistically significant (*p* > 0.05) (Figure 4).

#### 3.1.3. SQI of Forest Stand Types

**Figure 5:**
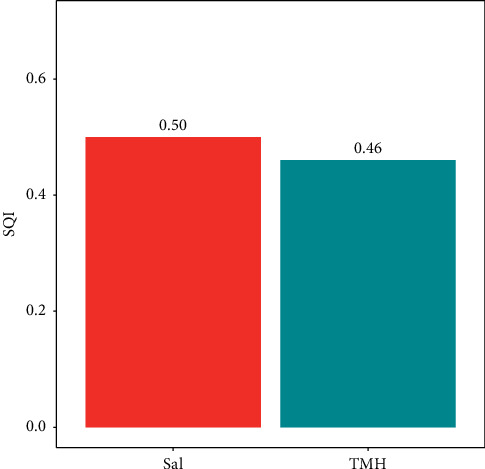
Soil Quality Index (SQI) values representing two forest types.

Using a common soil parameter approach, the SQI for Sal forest and TMH forest soils was found to be 0.50 and 0.46, respectively (Figure 5).

### 3.2. By Forest Management Regimes

#### 3.2.1. Soil Characteristics of Different Management Regimes

**Figure 6:**
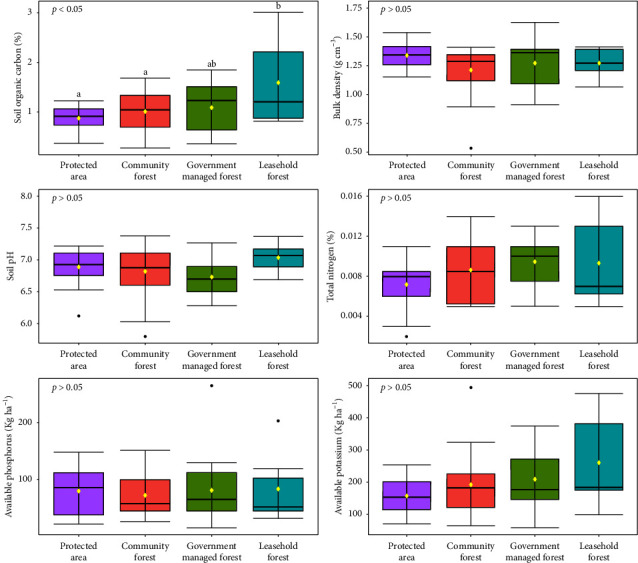
Soil properties of different management regimes. Different small letters indicate significant differences; yellow dot represents mean value.

Mean SOC was highest in leasehold forests (1.59%), followed by government-managed forests (1.09%), community forests (1.01%), and protected areas (0.87%) (Figure 6). BD was similar in government-managed forests (1.27 g·cm^−3^), leasehold forests (1.27 g·cm^−3^), and protected areas (1.27 g·cm^−3^), while it was slightly lower in community forests (1.21 g·cm^−3^) (Figure 6). Soil pH levels vary, with leasehold forests being slightly alkaline (pH 7.04), community forests, protected areas, and government-managed forests being slightly acidic (pH 6.82, 6.89, and 6.73, respectively) (Figure 6).

Total N remains consistently low at 0.01% across all regimes (Figure 6). Available P was highest in leasehold forests at 83.10 kg·ha^−1^, followed by government-managed forests (81.01 kg·ha^−1^), community forests (72.05 kg·ha^−1^), and protected areas (79.61 kg·ha^−1^) (Figure 6). Available K was found highest in leasehold forests (259.70 kg·ha^−1^), followed by government-managed forests (208.00 kg·ha^−1^), community forests (191.64 kg·ha^−1^), and protected areas (156.46 kg·ha^−1^) (Figure 6). Our study found significant differences (*p* < 0.05) only in SOC among the examined soil properties. Post-hoc analysis revealed that protected areas and community forests had similar SOC, while government-managed forests showed no significant difference with other regimes. Leasehold forests exhibited significantly higher SOC than all other regimes (Figure 6).

#### 3.2.2. SOC Stock of Different Management Regimes

Highest SOC stock was found in leasehold forest (56.74-ton ha^−1^) followed by government-managed forest (39.79 ton·ha^−1^) and community forest (37.17 ton·ha^−1^), whereas lowest SOC stock was found in protected area (34.54 ton·ha^−1^) (Figure 6).

**Figure 7:**
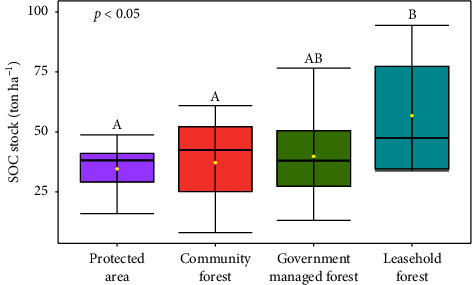
SOC stock in different forest management regimes. Different small letters indicate significant differences; yellow dot represents mean value.

Our study found significant differences (*p* < 0.05) in SOC stock between the forest management regimes. Post-hoc analysis revealed that protected areas and community forests had similar SOC stock, while government-managed forests showed no significant difference with other regimes. Leasehold forests exhibited significantly higher SOC stock than all other regimes (Figure 7).

#### 3.2.3. SQI of Different Management Regimes

**Figure 8:**
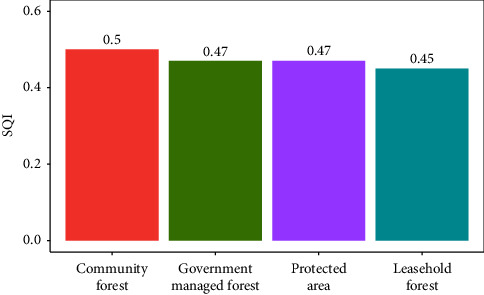
Comparison of soil quality index different management regimes.

Among different forest management regimes, community forest (0.50) had highest SQI followed by government-managed forest (0.47) and protected areas (0.47) whereas leasehold forest (0.45) had least SQI (Figure 8).

### 3.3. Relationship Between Environmental Factors and Soil Properties

**Figure 9:**
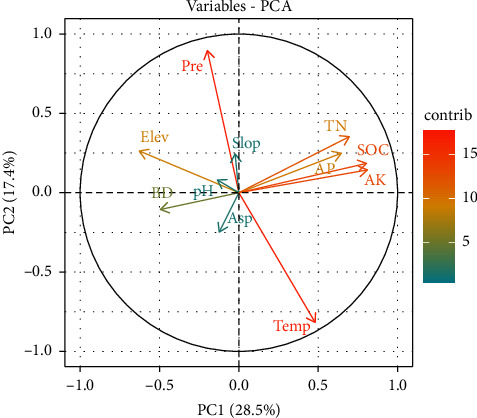
PCA of environmental factors and soil properties. Abbreviations: AK, available potassium; AP, available phosphorus; Asp, aspect; BD, bulk density; Elev, elevation; Prec, precipitation; Slop, slope; SOC, soil organic carbon percentage; Temp, temperature.

PCA was used to study the correlations between soil properties and environmental factors (climatic and topographic factors). Contributions from all factors were 28.5% for PC1 and 17.4% for PC2 (Figure 9). Precipitation and temperature had a highest contribution in the variability of soil properties.

## 4. Discussion

### 4.1. By Forest Stand Types

#### 4.1.1. Soil Characteristics of Forest Stand Types

The soil texture in both Sal and TMH forests was sandy loam and silty clay loam type, which falls in good and best soil rating (Table 2). This suggests that the texture of this forest can sustain appropriate growth of plants due to nutrients availability in finer soil and root respiration and by providing mechanical strength [81]. Our result coincides with the findings of Sigdel [82] in Royal Chitwan National Park (RCNP) and Thapa et al. [81] in Mixed Sal Forest in Chitwan district of central Nepal and Paudel and Shah [83] in tropical Sal Forest in Udayapur district of Eastern Nepal. A study of Bajracharya et al. [74] and Paudel and Shah [83] in tropical Sal forest reported the similar texture as suitable for good Sal regeneration and high-quality trees [84]. These textures are particularly abundant in the Terai, Siwalik, and Doon valleys, which might be because of the similar climate, parent materials, and kind of forest vegetation, i.e., Sal-dominated forest [81].

Mean SOC content in Sal forest was found lower (1.02%) than TMH forest (1.09%) (Figure 3) which is in line with Ghimire et al. [35] and Kafle [85], in Sal-dominated forest in Chitwan and Makwanpur districts of Central Nepal, respectively. Higher SOC content in TMH might be due the fact that majority of TMH forest plots were located protected areas and government-managed forests. Protected areas and government-managed forests, governed by regulations restricting human disturbance and deforestation, help preserve and accumulate organic matter in the soil, preventing soil degradation [12].

Most of the soils in Terai region are found to be acidic in nature [86]. However, the soil of Sal forest was found to be slightly more acidic than that of TMH forest (Figure 3). This might be probably due to the higher number of Sal trees and their saplings that leads to increased accumulation of leaf litter [87]. The pH range in the present study (6.82–6.85) was higher than the values reported in RCNP [82], Koshi Tappu Wildlife Reserve [88], tropical Sal Forest in Udayapur district [83], Sal Forest in Dhading [89], Mixed Sal Forest in Chitwan district [81], and Sal forest in India [90, 91]. A study by Ghimire et al. [35] in Sal forest of chure region of Makwanpur district has reported higher pH value (7.5), whereas Kharal et al. [89] in Sal forest of Dhading district have reported lower pH (4.74) than our study. This may be due to local environmental factors such as rainfall and vegetation composition [81, 83, 91]. Our studied forest are suitable for regeneration due to low pH because soils with higher pH generally have poorer capacity for regeneration [92] and vice versa [83, 87].

Our study found medium K ratings in both forests [76]. The soil in the Sal forest had higher K content than TMH forest (Figure 3). Das [90] also reported medium K content ranging from 181 kg ha^−1^–234 kg ha^−1^in Sal forest in India. Similarly, Paudel and Shah [83] reported higher K content in both pure Sal forest (267.73 kg ha^−1^) and Sal mixed forest (233.86 kg ha^−1^) in Udayapur district than our study. However, Ghimire et al. [35] found lower K content in Sal dominated forest (155.44 kg ha^−1^) than our study. According to Kumar et al. [91], a higher proportion of K was discovered to be responsible for the good prospering of Sal regeneration. Consequently, K content is crucial for the seed germination in Sal Forest [87].

This study had found high P ratings in both forest types, comparing to the soil fertility rating system [76]. The soil in the Sal forest had higher P content than the TMH forest soil (Figure 3). Our result is quite similar to the findings of Paudel and Shah [83] where they reported 76.64 kg·ha^−1^ P in pure Sal forest and 79.29 kg·ha^−1^ P in Sal mixed forest in Udayapur district, Nepal. However, Ghimire et al. [35] reported very low level of P (9.77 kg·ha^−1^) content in Sal forest of Chure region in Makwanpur district. Similarly, in a study by Das [90], the available P in the Sal forest of Jungle Mahal, India, was found to be in range of 36 kg·ha^−1^–74.2 kg·ha^−1^. Phosphorus availability increases tree growth, which strongly correlates with the establishment of forests [90]. Phosphorus availability is influenced by climatic conditions [93], organic matter content [35, 91], soil pH [94], and age of soil and land management practices [35]. A higher soil pH and a high value of organic matter in the forest may be responsible for the increased availability of phosphorus in pure Sal forest [95].

The total nitrogen levels in Nepal ranged from 0.05% to 0.40% [43]. However, total N level in both forest types in our study was 0.01% (Figure 3) which is low according to the scoring value by NARC [76]. This value was lower than the finding of Paudel and Sah [83] in tropical Sal forest in Udaypur district, Ghimire et al. [35] in Sal forest in Makwanpur district, Kharal et al. [89] in Sal Forest Dhading, and Thapa et al. [81] in mixed Sal forest in Chitwan district of Nepal. Similarly, a study by Kumar et al. [91] in Hazaribag, India, found 0.25% N in Ichak Sal forest and 0.26% N in Bishnugarh Sal forest which is higher than our study. This difference can be attributed to different factors such as vegetation uptake, organic matter decomposition, leaching, forest type, soil characteristics, and the absence of nitrogen-fixing plants [96]. Availability of P can also influence N levels by simulating N loss as N_2_O although it reduces N loss through leaching [96].

BD was found to be 1.268 g·cm^−3^ and 1.266 g·cm^−3^ in Sal forest and TMH forest, respectively (Figure 3), which align with Ranabhat et al. [97] in Nepalese forest soils. A study by Thapa et al. [81] in mixed Sal forest also found the similar bulk density range of 1.11–1.33 g·cm^−3^. Similarly, Kafle et al. [98] in Parsa National Park also reported similar bulk density (1.24 g·cm^−3^). However, Gautam and Mandal [99] in moist tropical Sal forest of Sunsari District reported slightly higher bulk density of 1.41 g·cm^−3^. Bajracharya et al. [74] also reported slightly higher bulk density (1.34 g·cm^−3^) in forest soil of watershed in Kavre district. This slight difference in bulk density in different study sites might be due to difference in organic matter content [81]. In general, organic matter (humus) content in soil can alters the BD, i.e., the higher the organic matter content, the lower the BD [81, 100].

The availability of nutrients in the soil is vital not only for plant growth and soil fertility but also constitutes a crucial parameter for the development of the Sal forest [101]. Deficiencies in nutrients such as total N, available P, and available K can manifest in noticeable symptoms, including premature defoliation, reduced leaf size, a slender taproot, and sluggish shoot growth [102]. In our study site, available P had a high rating, available K had a medium value, and total N had a low rating value based on the NARC [76] soil fertility rating system. According to Paudel and Shah [83], the Sal forest contained richer soil nutrients overall than the TMH forest, most likely because of the tree cover's increased intake of organic matter.

#### 4.1.2. SOC Stock of Forest Stand Types

SOC stocks vary with forest types, management regime, climate, composition of species, edaphic conditions, age, and disturbances [103]. One of important aspect that can affect SOC stocks within the forest is composition of species. Prior researches have confirmed that there are functional connections between plant diversity and carbon storage [104–108]. Forest stands with a higher prevalence of multiple species exhibit greater carbon storage that suggests a correlation. Ecosystems with diverse plant assemblages tend to demonstrate higher resource use efficiency, leading to increased productivity, litter production, and SOC content compared to stands dominated by a single species [109, 110]. This might explain the higher SOC stock in TMH forest than pure Sal forest in our study (Figure 4). Our finding aligns with Pradhan et al. [111] where they reported lower SOC stock in pure Sal forest than Pine-Shorea forest (88.54 ton·ha^−1^) and Schima-Castonopsis forest (43.94 ton·ha^−1^) in Pokhare Khola subwatershed of Dhadhing, Nepal. Similarly, SOC stock value of pure Sal forest in our study is lower than upper (104.4 ton·ha^−1^) and lower (62.5 ton·ha^−1^) mixed hardwood forest in Shivapuri Nagarjun National park, Nepal [47]. Variations in forest stand types can result in distinctions in both the quality and quantity of litter generated, potentially influencing litter decomposition rates and subsequently impacting SOC stocks [105]. Furthermore, Gairola et al. [112] revealed a positive correlation between SOC stock and total N, available P, and available K. The higher content of total N, available P, and available K observed in the TMH forest than pure Sal forest (Figure 4) may explain the lower SOC stock in the Sal forest.

#### 4.1.3. SQI of Forest Stand Types

In accordance with the criteria established by Bajracharya et al. [74], the SQI is classified as very poor if the ranking value falls below 0.2, poor for values ranging between 0.2 and 0.4, fair for values within the 0.4 to 0.6 range, good for values spanning from 0.6 to 0.8, and best for values between 0.8 and 1. The soil quality evaluation for both Sal forests and TMH forest yielded a fair rating 0.5 and 0.46, respectively (Figure 4). However, Sal forest has a higher SQI compared to the TMH forest. Sal trees are known to produce a large amount of leaf litter and other organic materials, which contributes to the accumulation of soil organic matter and supports soil microbial activity [83]. The dense canopy of the Salt forest can also help to reduce soil erosion by intercepting rainwater and reducing the impact of raindrops on the soil surface [113]. In contrast, the TMH forest is characterized by a more open canopy, with a lower density of trees and greater exposure to direct sunlight [114]. This can lead to higher rates of soil erosion, reduced soil organic matter buildup, and lower nutrient availability [115]. These factors may have contributed to the differences in SQI values observed for both forest types.

### 4.2. By Management Regimes

#### 4.2.1. Soil Characteristics

Our finding shows that leasehold forest has higher mean SOC % followed by government-managed forest, community forests, and protected areas (Figure 6). The reason for higher SOC in the both leasehold and government-managed forests is due to the strict rules and is undisturbed by human activity (where human interference is limited) than disturbed forests (where human interference is allowed) [27, 116, 117]. The lower SOC % in community forests than leasehold and government-managed forests is due to the thinning, pruning, clearance of leaf litter, cutting, and logging of the trees for the livelihood sustainability of the local people, as well as the disturbance from cattle grazing inside the community forest [28]. However, Gurung et al. [27] result contrast with our finding showing higher density of C in community forest than in government-managed forest which could be due to effective enforcement mechanisms in place in community forest to protect forests. Forests of protected areas had low SOC % (Figure 6) which could be an indication of the need for proper silvicultural treatments and management activities [118]. Furthermore, there was no significant difference in BD across different forest management regimes (Figure 6). However, a trend toward slightly higher BD values was observed in government-managed forests, leasehold forests, and protected areas, likely due to a combination of low organic matter content and coarse, sandy soil textures [119]. Also, livestock and wildlife population which together with farming activities might have had an impact on the soil structure thus increasing the BD in leasehold and government-managed forest [120]. The lower BD in community forests might be due to the low compaction of soil layer and high infiltration rate [121]. Soil pH levels vary, with leasehold forests being slightly alkaline, community forests, protected areas, and government-managed forests being slightly acidic (Figure 6). The acidic nature in above mentioned forest might be due to the acidifying properties of organic matter, aluminum, carbon dioxide, and presence of very low quantities of clay minerals [122]. Moreover, a study in Nigeria found relatively acidic nature of the soils could also be attributed to the high rainfall resulting in the leaching of some basic cations especially calcium from the surface horizons of the soils [123], whereas alkaline nature of leasehold forests might be due to the presence of calcium carbonate from a calcareous parent geological material [124]. Furthermore, a study in Mara River Basin, between Kenya and Tanzania, found relatively high soil pH at Ngerende sampling site due to low organic matter input in the grazing fields and probably accumulation of bases resulting from a compromised hydraulic conductivity that results from minimum leaching of the soluble bases [120]. Total N is low across all regimes which might be due to difficulty in measurement of Total N in soils and is difficult to interpret because levels of N are susceptible to change with storage of time, temperature, and moisture content [125]. This is similar to the study of Matano et al. [120] in the Silibwet sampling site in Mara River Basin stating low N is due to crop uptake as well as additional loss through food crop harvests or when vegetation is uprooted during land preparation. Cultivation of trees, in case of leasehold forest, for example, diminishes soil carbon within a few years of initial conversion and substantially lowers mineralizable N [126]. The concentration of P in soil of different forest regimes varies from 79.61 kg·ha^−1^ to 83.10 kg·ha^−1^ which was more than in Asia by [127] (11 kg·ha^−1^–25 kg·ha^−1^), Central Amazon by [128] (0.2 kg·ha^−1^–1.5 kg·ha^−1^) but less than in Halol range in Gujarat by [129] (113.92 kg·ha^−1^). The observed values clearly mentioned the high availability of phosphorus as all the values lies above (30 kg·ha^−1^) in Nepal [130] and (> 24.6 kg·ha^−1^) in India [131]. The high concentration of P in leasehold forests (Figure 6) is likely due to the establishment of tree-based traditional agroforestry systems in degraded land, which significantly enhances soil biological activity [132]. Little to no agricultural activities inside these forest types is also the main reason of having high P which is similar to the study of Christensen et al. [133] in the Minnesota River Basin, United States. The concentration of K under different forest management varies from 156.46 kg·ha^−1^–259.70 kg·ha^−1^ which was parallel to the study in India by [131] (< 108 kg·ha^−1^) but high comparison with the study in Asia by [127] (0 kg·ha^−1^–50 kg·ha^−1^) and in central Amazon by [128] (0.3 kg·ha^−1^–2.8 kg·ha^−1^). The cultivation of multipurpose trees in leasehold forests and community forests is the primary cause for the higher K accumulation, which is consistent with the findings of Ramesh et al. [134] in Meghalaya, India. In addition, the high concentration of K in Terai soil is due to the presence of potassium containing minerals such as illite, muscovite, gluconite, biotite, phlogopite, sanidine, and orthoclase [135]. The K content is high in case of community forests, protected areas, and government-managed forest because of the acidic nature of soil (pH = less than 7) which is parallel to the study of [136] in the Kedarnath Wildlife Sanctuary, Western Himalaya (India). Gupta and Sharma [137] and Raina and Gupta [138] in Uttarakhand, India, also asserted that available K is not significantly affected by soil organic matter, as it is not the direct source of K, which aligns with the findings of our study.

#### 4.2.2. SOC Stock

Our study showed that SOC stock rates vary significantly among the forests under different management regimes (Figure 6). This result is in line with the results of the study by Gurung et al. [27] from Terai Arc Landscape (TAL). Mean SOC stock was highest in the leasehold forests followed by government-managed forests, community forests, and protected areas (Figure 6). This could be the adoption of agroforestry as some portion of land within leasehold forest is leased user groups or individuals (often poorer households) for rehabilitation and productive use [139]. Another probable reason could be due to fewer sample size across management regimes such as leasehold forest. Similar findings have been reported by Baral et al. [140] and Oli and Shrestha [141] in different forest of Nepal regarding the high SOC content in leasehold forests of the Terai region. On the contrary, some studies [139, 142] found higher SOC level in community forests than leasehold forests which may be because of less sand content in the CF soil than in the leasehold forest soil. In addition, the low SOC content in protected areas is likely due to the more humid and more acidic soil conditions, which are unfavorable for the complete mineralization of organic residues. This finding is consistent with the results of a study by Šlepetienė et al. [143] in EU protected areas and Krekenava regional park.

#### 4.2.3. SQI

Our study found that CF have highest SQI than other forest types (Figure 8). The study by Thapa et al. [81] supports the findings of the current study, which found that Kankali CF in Chitwan, Nepal, has higher soil SQI. This is likely due to the regulation of leaf litter collection and the adoption of appropriate silvicultural operations in community forests, which can help to improve soil health [81]. The SQI value under the study of Ghimire et al. [35] in the Chure region of Central Nepal was good in case of forest soil (0.82) followed by 0.66 (Fair) of bari, 0.64 (Fair) of khet, and 0.40 (Poor) of degraded land. However, it is contrast with the finding of Kalu et al. [37] in Panchase area of western Nepal. Kalu et al. [37] showed SQI to be significantly higher in the protected forest (0.95), followed by the community forests (0.91), pasture (0.88), khet (0.81), and bari (0.79). The low SQI in leasehold forest is due to the high dependency of poor peoples in the forest products extraction due to insecure exclusive use rights resulting in soil erosion [144]. Like our study, Lal [145] reported that SOC to be a key attribute of soil quality because it determines physical, chemical, and biological soil properties. N, P, and K are also the most important parameters to determine the soil quality in this study which is consistent to the study of Thapa et al. [81] in central Nepal.

### 4.3. Relationship Between Environmental Factors and Soil Properties

In our study carried out in the southern belt of Nepal, precipitation and temperature were the primary factors influencing soil property variability (Figure 9). Both forest stand types observed similar average annual temperature of 25 degree Celsius with maximum temperature up to 45-degree Celsius. However, there is difference in precipitation, with the eastern Nepal receiving higher precipitation (average annual precipitation of 1500–2000 mm·yr^−1^) in comparison to the western Nepal (average annual precipitation of 1100–1400 mm·yr^−1^). Feng et al. [146] discussed positive relationship of precipitation with SOC in different land uses including different forest types. This is because the adequate rainfall supports vegetation growth and microbial activity, enhancing soil nutrients and organic carbon by incorporating organic matter [147, 148]. Moreover, the precipitation also effects nitrogen transformation and availability as it enhances nitrification and mineralization rates of soil nitrogen [149, 150]. The total P concentrations are significantly correlated with the precipitation intensity due to the more dissolved phosphorus with increasing amount of rainfall [151]. However, the soil K and soil pH are negatively affected by precipitation due to downward movement of dissolved potassium through soil profile and increased acidification due to high level of leaching caused by higher precipitation, respectively [148, 151, 152].

The increased evaporation due to higher temperatures reduces plant productivity, leading to similar and low level of SOC inputs in both forest stands and management regimes [153]. We found the low-level SOC (around 1%) which might be due to the high average temperature of the Terai region that accelerates organic carbon breakdown, negatively impacting SOC [153]. The low level of N content (around 0.01%) in the Terai region could be due to the higher annual average temperature leading to the nitrogen depletion through leaching, denitrification, and volatilization as soil temperature rises in the region [154, 155]. In general, higher temperatures in forests could also increase available P content due to enhanced microbial breakdown processes [156] which is line with our finding of available P range of around 60–80 kg/ha in both forest stand types. Tian et al. [156] also observed such relation higher P concentrations in forests, as microorganisms decompose organic matter and release P into the soil. In addition, as temperatures increase, soil pH tends to rise due to the denaturation of organic acids, particularly at high temperatures [157]. Both forests stand types exhibited the higher range of K availability (180–200 kg·ha^−1^) which is due to the high level of weathering of K-containing minerals in higher temperatures, contributing to higher K availability in the soil [158].

Despite these influences of precipitation and temperatures on soil dynamics, our study did not find any significant differences in terms of soil properties and SOC across two forest stand types, likely due to similar climatic conditions throughout the study area. Other factors such as aspect, elevation, and slope (Figure 9) contributed minimally to soil variability across the forest stand types, depicting the similar topography in the Terai region.

### 4.4. Implication for the Study

The findings of this study offer valuable insights into the importance of soil quality for the sustainability of forest ecosystems, especially in the context of sustainable forest management (SFM). SFM aims to balance ecological, socioeconomic, and institutional factors to maintain and enhance forest resources [159]. Among these, soil health is a critical ecological component that underpins forest productivity, biodiversity, and resilience. Soil management plays a pivotal role in improving habitat quality and supporting diverse forest ecosystems. Our results demonstrate that soil fertility management can significantly contribute to sustainable forest outcomes, particularly in the context of the Terai region, which faces specific challenges such as seasonal waterlogging during the rainy season. This waterlogging disrupts nutrient availability, depletes soil quality, and consequently hampers forest regeneration efforts [160]. Our findings highlight the need for targeted soil management interventions to address these issues, particularly the highly acidic soils and low nitrogen levels observed in our study. These results have important implications for forest policy and SFM guidelines. According to the SFM guidelines and the Scientific Forest Management Policy, soil management is prioritized, particularly in areas undergoing regeneration felling [161]. The guidelines emphasize that soil quality must be maintained or improved to ensure the long-term sustainability of forest resources. Therefore, our study suggests that soil management should be a key focus in developing and implementing policies aimed at enhancing forest productivity and ecological resilience. Considering our findings, we recommend that the government and relevant stakeholders invest in further research and monitoring of soil conditions in the Terai forests, with a focus on mitigating waterlogging and improving nutrient availability. Such efforts would not only support forest regeneration but also align with broader national and global objectives for sustainable land management and biodiversity conservation.

### 4.5. Study Limitation and Research Recommendations

Fewer sample size per forest management regime is our primary limitation as larger sample size would have increased the statistical power of the study, allowing for more robust conclusions. In this study, parent materials were not included as the environmental which could affect the soil parameters and soil quality. Our study was restricted to the 0–30 cm soil depth, which may not fully capture variations in soil quality and carbon storage that occur at greater depths. Therefore, future studies should assess soil quality across a broader depth profile, including deeper layers up to 1 m. Similarly, this research, conducted under a domestic institution with limited resources, was constrained in the number of soil quality indicators that could be assessed. A more extensive study would allow for the evaluation of a broader array of soil quality indicators, offering a more complete understanding of soil health dynamics in forest ecosystems. For example, incorporating additional chemical, biological, and physical parameters, such as microbial biomass, enzyme activities, and soil aggregation, would yield deeper insights into soil functionality and resilience in different forest stand and management regimes. Our research was conducted at a single point in time, which limits our ability to assess seasonal and long-term changes in soil quality and carbon dynamics. Therefore, longitudinal studies would provide insights into temporal variations in soil quality and carbon stocks, especially under changing climatic conditions.

## 5. Conclusion

This study was conducted to assess SOC stock and soil quality across different forest stand types and management regimes. Both forest types exhibited almost similar carbon storage capacities in soil. Most of the soil quality indicators were not significantly influenced by different forest stand types and management regimes. However, Sal forest (0.50) was found to be superior than TMH forest (0.46) with higher SQI. Unexpectedly, leasehold forest was found to store higher SOC stock (56.74 ton·ha^−1^) than other management regimes, which could be due to small sample size. However, community forest had superior soil quality (0.50) than other regimes because of better management activities by local communities (forest user groups) assisted by forest officials. Addressing lower soil quality in other management regimes may help improve soil conditions through targeted practices. Precipitation and temperature emerged as key drivers that influenced soil properties in the forest types and management regimes. Therefore, information can be essential for predicting and managing forest soil responses to climate change, land use practices, and other anthropogenic disturbances.

## Figures and Tables

**Table 1:** Detail information of plots in this study.

District	Forest Type	Lat	Lon	Prec	Temp	Asp	Elev	Slp	SOC Stock	BD	TN	pH	AK	AP	SOC	Regimes
Parsa	TMH	27.25	84.71	1691.58	23.38	90	159	2	45.02	1.503	0.008	6.93	242.59	132.72	1	PA
Parsa	S	27.25	84.71	1924.77	22.35	270	164	1	48.7	1.343	0.009	7.22	213.61	108.4	1.21	PA
Parsa	S	27.25	84.71	1924.77	22.35	246	162	2	28.59	1.315	0.008	6.53	187.76	86.14	0.72	PA
Parsa	S	27.25	84.79	1924.77	22.35	0	170	0	29.51	1.359	0.008	6.68	124.45	115.1	0.72	PA
Parsa	TMH	27.25	84.79	1924.77	22.35	132	176	3	38.14	1.309	0.008	6.68	140.45	131.35	0.97	PA
Kanchanpur	S	28.80	80.46	1707.69	22.07	180	218	2	25.85	1.39	0.007	6.7	140.45	14.63	0.62	GMF
Kanchanpur	TMH	28.80	80.46	1707.69	22.07	90	216	3	21.08	1.39	0.007	6.7	153.15	112.42	0.51	GMF
Bara	TMH	27.21	85.03	1400.20	23.58	90	200	1	53.68	1.616	0.011	6.72	149.45	47.6	1.11	GMF
Bara	TMH	27.22	85.03	1400.20	23.58	253	206	3	76.49	1.625	0.012	7.15	154.96	41.11	1.57	GMF
Bara	S	27.10	85.11	1400.20	23.58	107	130	3	59.1	1.329	0.007	6.84	179.93	50.28	1.48	LF
Bara	TMH	27.14	85.15	1400.20	23.58	270	148	3	62.53	1.393	0.01	6.7	306.58	72.44	1.5	GMF
Kanchanpur	S	28.76	80.50	1918.88	20.73	150	209	2	38.01	1.439	0.009	6.42	137.49	54.41	0.88	GMF
Rautahat	TMH	27.07	85.39	1688.79	22.68	0	119	6	94.4	1.066	0.015	7.04	475.95	203.3	2.95	LF
Rautahat	TMH	27.07	85.40	1688.79	22.68	294	121	2	83.35	1.219	0.016	6.69	446.7	118.68	2.28	LF
Rautahat	TMH	27.07	85.40	1688.79	22.68	132	121	3	33.76	1.201	0.007	7.2	185.8	53.07	0.94	LF
Sarlahi	TMH	27.03	85.51	1323.26	25.07	159	124	3	41.22	0.918	0.01	6.43	274.04	116.93	1.5	GMF
Sarlahi	TMH	27.03	85.51	1323.26	25.07	312	122	3	35.82	1.012	0.01	6.7	139.99	78.6	1.18	GMF
Sarlahi	S	27.03	85.52	1323.26	25.07	0	122	0	37.04	0.973	0.012	6.57	370.2	126.4	1.27	GMF
Sarlahi	TMH	27.03	85.52	1323.26	25.07	228	126	1	46.66	1.095	0.011	6.4	268.91	111.59	1.42	GMF
Kailali	TMH	28.84	80.58	1918.88	20.73	294	212	2	25.52	1.033	0.005	7.24	62.9	44.41	0.82	CF
Siraha	TMH	26.88	86.20	1536.85	23.59	180	126	4	14.97	0.537	0.013	6.64	99.05	28.13	0.93	CF
Ilam	TMH	26.75	87.24	1756.93	22.80	277	163	7	50.62	1.108	0.011	6.28	245.23	129.62	1.52	GMF
Ilam	S	26.75	87.24	1756.93	22.80	90	158	4	56.17	1.108	0.011	6.03	212.22	97.48	1.69	CF
Morang	TMH	26.71	87.32	1756.93	22.80	270	153	1	48.48	1.213	0.013	7.13	262.43	53.17	1.33	CF
Morang	S	26.71	87.40	1756.93	22.80	114	193	2	53.35	1.113	0.014	7.28	323.3	100.57	1.6	CF
Morang	S	26.71	87.40	1756.93	22.80	90	194	8	54.72	1.29	0.014	7.05	286.27	50.8	1.41	CF
Morang	S	26.67	87.52	2061.91	22.04	180	174	4	50.21	0.912	0.011	7.27	374.17	265.12	1.84	GMF
Morang	TMH	26.67	87.52	2061.91	22.04	90	161	5	13.99	1.087	0.01	7.05	326.86	31.63	0.43	GMF
Ilam	TMH	26.73	87.72	2061.91	22.04	197	229	11	20.66	1.186	0.01	6.45	97.44	43.68	0.58	CF
Kailali	TMH	28.77	80.79	2527.40	18.80	311	216	1	58.45	1.362	0.006	6.95	197.05	44.2	1.43	GMF
Jhapa	S	26.51	88.04	2675.49	20.95	132	108	3	44.29	1.263	0.01	6.33	204.69	55.63	1.17	CF
Jhapa	S	26.51	88.04	2675.49	20.95	90	91	1	33.8	1.091	0.012	7.25	104.83	44.51	1.03	CF
Kailali	S	28.70	80.87	1644.49	22.48	66	187	2	29.98	1.318	0.01	6.72	148.86	43.9	0.76	GMF
Kailali	S	28.66	80.91	1644.49	22.48	150	183	2	46.92	1.362	0.013	6.69	224.85	104.26	1.15	GMF
Kailali	S	28.67	80.91	1644.49	22.48	143	180	3	28.2	1.524	0.007	6.85	175.72	64.81	0.62	GMF
Kailali	TMH	28.59	80.95	1644.49	22.48	294	166	2	26.18	1.37	0.008	6.34	106.98	33.49	0.64	GMF
Bardiya	S	28.42	81.29	1414.51	23.24	90	174	3	25.01	1.372	0.008	6.56	188.83	58.53	0.61	CF
Bardiya	S	28.46	81.33	1414.51	23.24	300	182	4	41.57	1.243	0.002	7.15	90.16	38.22	1.11	PA
Bardiya	S	28.38	81.37	1414.51	23.24	66	186	2	42.95	1.204	0.01	6.92	154.77	94.59	1.19	PA
Kanchanpur	TMH	28.90	80.13	1716.05	22.57	0	200	3	23.16	1.152	0.011	6.83	69.22	30.29	0.67	PA
Bardiya	TMH	28.28	81.62	1975.84	21.84	0	192	0	34.81	1.268	0.006	7.14	181.84	36.47	0.92	PA
Bardiya	TMH	28.28	81.62	1975.84	21.84	131	186	3	18.17	1.379	0.003	7.18	112.36	32.56	0.44	PA
Bardiya	TMH	28.28	81.62	1975.84	21.84	229	195	1	30.44	1.363	0.006	6.12	113.93	59.14	0.74	PA
Banke	TMH	28.28	81.70	1975.84	21.84	180	241	5	40.26	1.485	0.006	7.02	151.87	20.91	0.9	PA
Banke	TMH	27.99	81.78	1174.15	23.95	229	153	1	54.53	1.407	0.006	5.8	164.64	34.21	1.29	CF
Banke	TMH	28.00	81.78	1174.15	23.95	311	148	1	43.49	1.409	0.005	7.38	213.52	52.02	1.03	CF
Banke	S	27.92	81.83	1174.15	23.95	311	156	1	55.6	1.302	0.008	6.84	494.49	80.78	1.42	CF
Banke	S	27.92	81.83	1174.15	23.95	131	152	3	43.96	1.339	0.005	6.86	228.7	109.63	1.09	CF
Banke	S	28.07	81.87	1469.52	22.52	219	175	8	23.48	1.327	0.005	7	146.6	108.4	0.59	CF
Kapilvastu	S	27.68	82.72	1315.89	23.27	312	153	1	33.96	1.413	0.006	7.1	172.27	31.02	0.8	LF
Kapilvastu	TMH	27.68	82.73	1315.89	23.27	270	157	2	35.86	1.411	0.005	7.37	97.57	42.24	0.85	LF
Kanchanpur	S	28.90	80.25	1707.69	22.07	229	211	6	15.95	1.452	0.007	6.88	83.7	59.66	0.37	PA
Kanchanpur	TMH	28.90	80.25	1707.69	22.07	198	211	7	40.45	1.536	0.007	6.99	226.73	100.77	0.88	PA
Kapilvastu	TMH	27.72	82.89	1363.09	23.27	0	159	1	13.08	1.251	0.005	7.27	56.97	45.95	0.35	GMF
Kapilvastu	TMH	27.68	82.97	1363.09	23.27	114	136	2	27.17	1.345	0.006	7.3	117.2	25.55	0.67	CF
Kapilvastu	S	27.72	83.05	1486.16	23.16	180	155	1	8.01	0.894	0.005	6.71	131.04	124.86	0.3	CF
Kapilvastu	S	27.72	83.17	1486.16	23.16	228	167	6	28.36	1.358	0.006	6.9	138.3	32.56	0.7	CF
Rupendehi	S	27.65	83.49	1857.29	22.32	0	141	0	60.84	1.286	0.009	7.03	173.78	60.48	1.58	CF
Rupendehi	S	27.65	83.50	1857.29	22.32	180	141	3	41.48	1.32	0.011	6.9	251.55	143.92	1.05	CF
Rupendehi	S	27.65	83.50	1857.29	22.32	246	145	2	44.71	1.362	0.005	6.59	210.6	151.16	1.09	CF
Kanchanpur	S	28.76	80.34	1707.69	22.07	0	194	1	40.32	1.185	0.009	7.08	253.48	147.83	1.13	PA
Kanchanpur	S	28.90	80.37	1707.69	22.07	229	231	1	9.08	1.136	0.009	6.84	103.76	84.6	0.27	CF

**Note:**																
**Abb.**	**Full Form**	**Unit**														

Lat	Latitude	degree														
Lon	Longitude	degree														
Prec-	Annual average precipitation	mm														
Temp	Monthly average temperature	degree Celsius														
Asp	Aspect	degree														
Elev	Elevation	meters														
Slp	Slope	degree														
SOC Stock	Soil organic carbon stock	ton ha^−1^														
BD	Bulk density	g cm^−3^														
TN	Total nitrogen	%														
AK	Available potassium	kg ha^−1^														
AP	Available phosphorus	kg ha^−1^														
SOC	Percentage of soil organic carbon	%														
PA	Protected area															
GMF	Government-managed forest															
LF	Leasehold forest															
CF	Community forest															

**Table 2:** Common soil parameters and ranking values for SQI in Nepal.

	Ranking values				
Parameters	0.2	0.4	0.6	0.8	1
Soil textural class	C, S	CL, SC, SiC	Si, LS	L, SiL, SL	SiCL, SCL
Soil pH	< 4	4–4.9	5–5.9	6–6.4	6.5–7.5
SOC%	< 0.5	0.6–1	1.1–2	2.1–4	> 4
Fertility (NPK)	Low	Mod low	Moderate	Mod. High	High
SQR	Very poor	Poor	Fair	Good	Best

Source: [74].

**Table 3:** N, P, and K interpretation of soil of Nepal.

Total *N* (%)		Available P (kg/ha)		Exchangeable K (kg/ha)	
Range	Level	Range	Level	Range	Level
< 0.1	Low	< 31	Low	< 110	Low
0.1–0.2	Medium	31–55	Medium	110–280	Medium
> 0.2	High	> 55	High	> 280	High

Source: [76].

**Table 4:** Moran's test for spatial autocorrelation (*p* value) of soil properties.

Soil properties	Moran I statistics	Expectation	Standard deviation	*p* value
SOC (%)	0.28	−0.02	0.11	0.0055422
Bulk density	0.45	−0.02	0.11	2*E* − 05
SOC stock	0.26	−0.02	0.11	0.01295518
Total nitrogen	0.45	−0.02	0.11	3.03*E* − 05
Available phosphorus	0.03	−0.02	0.11	0.6951414
Available potassium	0.33	−0.02	0.11	0.001686012
pH	−0.03	−0.02	0.11	0.9121524

## Data Availability

Data will be made available on request.
